# The extrahepatic events of Asian patients with primary biliary cholangitis: A 30-year cohort study

**DOI:** 10.1038/s41598-019-44081-x

**Published:** 2019-05-20

**Authors:** Cheng-Yu Lin, Ya-Ting Cheng, Ming-Ling Chang, Rong-Nan Chien

**Affiliations:** 1 Liver Research Center, Division of Gastroenterology, Department of Gastroenterology and Hepatology, Chang Gung Memorial Hospital, Taoyuan, Taiwan; 2 Department of Medicine, College of Medicine, Chang Gung University, Taoyuan, Taiwan

**Keywords:** Hepatology, Health care

## Abstract

The extrahepatic complications of primary biliary cholangitis (PBC) in Asian patients remain elusive. A 30-year cohort study of 150 Taiwanese PBC patients treated with ursodeoxycholic acid (UDCA) was conducted. Patients with alkaline phosphatase levels >1.67 × ULN after 1-year treatment were considered suboptimal responders. At baseline, of 150 patients (mean age: 53.75 years), 128 (85.3%) were females, and 34 (22.8%) had cirrhosis. The cumulative incidences of various incident events were all-cause mortality or liver transplantation: 46.7%; extrahepatic mortality: 24.5%; extrahepatic malignancies: 8.1%; hypertension: 46.2%; dyslipidemia: 44.1%; diabetes: 30.6%; hyperuricemia: 11.2%; acute coronary syndrome: 3.1%; cerebral vascular accident (CVA): 8.9%; autoimmune diseases: 16%; and osteoporosis: 20.9%. The 5- to 20-year cumulative incidences for all-cause mortality or liver transplantation and extrahepatic mortality were 16.2–41.3% and 3.1–11.9%, respectively. Baseline associations were age and alpha-fetoprotein levels with extrahepatic mortality, 80% due to sepsis; age with extrahepatic malignancies and hypertension; gender and hyperuricemia with CVA; and UDCA response with autoimmune disease. Conclusions: Sepsis accounted for most extrahepatic mortality in PBC patients, and the longer the follow-up was, the higher the extrahepatic/all-cause mortality ratio. Baseline age is crucial for incident extrahepatic events and only CVA shows gender-dimorphism; the association between UDCA response and autoimmune disease requires further investigation.

## Introduction

Primary biliary cholangitis (PBC), formerly called primary biliary cirrhosis^1^, predominantly affects middle-aged women^2^. It is a rare, chronic progressive cholestatic liver disease characterized by the autoimmune-mediated destruction of the small- and medium-sized intrahepatic bile ducts^3^. Without treatment, most PBC patients eventually develop hepatic fibrosis and may require liver transplantation during the late stage of the disease^3^. Currently, ursodeoxycholic acid (UDCA) and obeticholic acid (OCA) are the two approved therapies for PBC^4^. However, some patients treated with UDCA or OCA do not exhibit adequate responses, as 30–40% of PBC patients do not respond to UDCA^5^ and up to 60% of UDCA suboptimal responder do not show therapeutic effect to OCA^6^.

In addition to hepatic manifestations such as liver cirrhosis and hepatocellular carcinoma (HCC)^3^, PBC can lead to multiple extrahepatic manifestations, including extrahepatic malignancies (EMs)^7^, autoimmune diseases^8^, dyslipidemia, and other metabolic diseases^9^. The prevalence of EM in PBC patients ranged from 4.5%^10^ to 15.7%^11^. Although PBC patients have a high incidence of HCC^12^, the risk of EM is even higher than that of HCC^13^. The onset of EM in PBC patients does not influence the natural history of their liver disease^7^. Moreover, antimitochondrial antibodies (AMAs) are detected in over 90% of patients with PBC^2,3^. The high frequency of AMA underlines not only its autoimmune process but also that PBC can occur in association with other autoimmune disorders^14^. Consistently, up to 1/3 of PBC patients have a concomitant extrahepatic autoimmune disease^15^. Common extrahepatic autoimmune conditions include Sjögren syndrome (SS), autoimmune thyroid diseases, and systemic lupus erythematosus (SLE)^8^. Although extrahepatic autoimmune diseases might be associated with EM development in PBC^7^, whether extrahepatic autoimmune diseases affect the prognosis of PBC patients remains conflicting^15,16^. In addition, PBC patients are prone to hypercholesteremia^2^. However, PBC is not associated with an increased risk of myocardial infarction, stroke, transient ischemic attack (TIA)^17^, and subclinical atherosclerosis^18^. The harmlessness of hypercholesteremia in cardiovascular risks of PBC is probably due to lower amounts of visceral fat^19^, higher adiponectin concentrations^20^, and more lipoprotein X^21^ and lipoprotein A1 particles^22^, compared with the controls. However, the impacts of hypercholesteremia on cardiovascular events in PBC patients may vary with various disease stages, as less advanced PBC with moderate hypercholesterolemia has been linked to an increased cardiovascular risk^23^. Moreover, after accounting for hepatic and cancer-related deaths, there is unexplained excess mortality associated with PBC^24^. Even for asymptomatic PBC patients, the overall survival was shorter than that predicted for an age- and gender-matched control population^25^.

Especially in Asia, the extrahepatic complications and associated mortality in PBC patients remain undetermined, and the situation is even more complicated when considering the UDCA suboptimal responders who are at high risk for serious complications^5^. Accordingly, we conducted a 30-year hospital-based cohort study to elucidate the extrahepatic manifestations and mortality as well as the associated factors of PBC in an Asian country, Taiwan.

## Methods

### Patients

Patients older than 18 years of age with a diagnosis of PBC were consecutively recruited at a Taiwan tertiary referral center between July 1988 and June 2018. The diagnosis of PBC was based on the following criteria: (1) an AMA positivity higher than 1:40, (2) abnormal alkaline phosphatase (Alk-P) levels [≥1.5 X upper limit of normal (ULN)] and/or a compatible liver histology, and (3) an AMA negative variant: abnormal Alk-P levels, and a liver histology compatible with PBC. The subjects with autoimmune hepatitis, human immunodeficiency virus, hepatitis B infection, hepatitis C infection, hemochromatosis, active alcohol consumption, primary cancer and recipients of solid organ transplants, and those with a follow-up <1 year or poor compliance to UDCA therapy with were excluded.

### Study design

As a retrospective study, the clinical details of each patient were included anonymously with a composite code number in a database that contained detailed information including baseline demographic data and symptoms, physical findings and laboratory results at presentation and at the time of diagnosis and referral, as well as data collected at subsequent 3-month routine clinical visits, and emergency and hospital admissions. Moreover, for each patient, the following were recorded: the dates of complications and treatment, data from the last follow-up, dates of liver transplantation and death, and the cause of death. Biochemistry tests including AMA, antinuclear antibody (ANA), aspartate aminotransferase (AST), alanine aminotransferase (ALT), total bilirubin, albumin, Alk-p, γ-glutamyl transpeptidase (γ-GT), alpha-fetoprotein (AFP) and total cholesterol (TC) were conducted on the fasting serum of the enrolled participants. Cardiac injury or function tests, including creatine kinase and B-type natriuretic peptide, and electrocardiograms were performed as indicated. The aforementioned profiles were measured using routine automated techniques at the clinical pathology or research laboratories of the hospital. Liver ultrasound was performed every 6 months. All enrolled PBC patients were treated with UDCA at a standard dose of 13–15 mg/kg/day. Patients whose Alk-P levels > 1.67 × ULN after 1 year of treatment were defined as suboptimal responders^26^. The diagnosis of liver cirrhosis was made by earlier histologic findings or ultrasonographic findings compatible with cirrhosis and supplemented with esophageal or gastric varices, splenomegaly and/or thrombocytopenia. The incidence and risk factors of extrahepatic manifestations, including diabetes, hypertension, hyperuricemia, dyslipidemia, acute coronary syndrome (ACS), cerebrovascular accident (CVA), autoimmune diseases [SS, SLE, systemic sclerosis, rheumatoid arthritis (RA), autoimmune thyroid disease and psoriasis], osteoporosis, extrahepatic cancer and mortality, were surveyed. Diabetes was defined as a fasting plasma glucose level ≥126 mg/dl, plasma glucose ≥200 mg/dl two hours after a 75 g oral glucose load as in a glucose tolerance test, symptoms of high blood sugar and casual plasma glucose ≥200 mg/dl, glycated hemoglobin ≥6.5% in the diabetes control and complications trial^27^, or the associated diagnosis of diabetes [International Classification of Diseases, Ninth Revision (ICD-9) code 250] with anti-diabetic agents noted based on chart review. Hypertension was defined according to three criteria: systolic blood pressure ≥140 mm Hg, diastolic blood pressure ≥90 mm Hg or the use of medication to control hypertension^28^ or the associated diagnosis of hypertension (ICD-9: 401.9). Dyslipidemia was defined by serum concentrations of TC ≥ 200 mg/dL, low-density lipoprotein-cholesterol levels ≥130 mg/dL, triglycerides ≥150 mg/dL^29^, the associated diagnosis of dyslipidemia (ICD-9:272.0~272.4) or the usage of lipid-lowering agents based on chart review. Hyperuricemia was defined by serum uric acid levels >7 mg/dL, the presence of monosodium urate crystals by aspiration and examination of synovial fluid^30^, the presence of an associated diagnosis (ICD-9: 274), or the use of associated medications, including colchicine and allopurinol, based on chart review. ACS was defined as the presence of unstable angina, non-ST elevation myocardial infarction, ST elevation myocardial infarction^31^, or the presence of an associated diagnosis (ICD-9: 411.1, 411.81, 410) based on chart review. The CVAs were defined as TIA, ischemic stroke and hemorrhagic stroke identified using the chart review (ICD-9: 430~437) and confirmed by a review of medical records/registries^32^. SLE was diagnosed based on the American College of rheumatology criteria^33^ or the presence of an associated diagnosis (ICD-9: 710.0) based on chart review. SS was diagnosed based on European-USA consensus criteria^34^ or the presence of an associated diagnosis (ICD 9: 710.2) based on chart review. RA was diagnosed based on the revised American College of Rheumatology/European League Against Rheumatism collaborative initiative criteria for RA^35^ or the presence of an associated diagnosis (ICD-9: 714.0 or 714.3) on chart review. Psoriasis was defined as the presence of an associated diagnosis (ICD-9: 696.0, 696.1,696.8) based on chart review. Osteoporosis was defined as the presence of an associated diagnosis (ICD-9: 733.00) from chart review. Systemic sclerosis was diagnosed as described previously^36^. The primary cancers were diagnosed based on pathology, confirmed by the specialists of each primary cancer, and the diagnosis and stage of each cancer were registered with the National Cancer Registration.

### Statistical analyses

All statistical analyses were performed using the Statistical Package for Social Science (SPSS package version 21, SPSS Inc., Chicago, IL, USA) software. Continuous variables were analyzed using Student’s t-test, and categorical variables were analyzed using a chi-square test or Fisher’s exact test as appropriate. Nonparametric tests were applied where indicated. The collinearity of variables was determined by linear regression tests. Kaplan-Meier or univariate Cox regression analyses were used to assess the relationship between various variables and patient events. Multivariate Cox regression models were used to assess the relationship between various dependent and independent variables by adjusting for all the independent variables with a *p* value < 0.05 in the univariate analyses. Receiver operating characteristic curve (ROC) analyses were performed to evaluate if independent variables were significant predictors of the dependent variables. Youden index was assessed to identify the optimum cut-off values of the independent variables from the coordinate points of the ROC curves^37^. Binary logistic regression analysis was performed to assess the variables that accounted for the emergence of events. Statistical significance was defined at the 5% level based on two-tailed tests of the null hypothesis.

### Institutional review board

The study was conducted in accordance with good clinical practice and all applicable regulations, including the Declaration of Helsinki and local regulatory requirements and was approved by the ethics committee of Chang-Gung Memorial Hospital. Informed consent was obtained from the participants.

## Results

### Baseline characteristics

A total of 150 consecutive patients with PBC were enrolled in the current study. The baseline characteristics are listed in Table 1. Of 150 patients, 128 (85.3%) were female, with a female-to-male ratio of 5.82:1 and a mean and median age of 53.75 and 53.00 years, respectively. Additionally, 34 (22.8%) patients had liver cirrhosis, 5 (3.4%) had varices, 10 (6.8%) had hypertension, 7 (4.8%) had dyslipidemia, 6 (4.1%) had diabetes, 3 (2.0%) had hyperuricemia and 7 (4.6%, 2 with SS, 2 with autoimmune thyroiditis, 1 with systemic sclerosis, 1 with both SS and autoimmune thyroiditis and 1 with both SS and systemic sclerosis) had autoimmune diseases. The female patients had higher AST/ALT ratios (*p* = 0.008) and cholesterol levels (*p* = 0.029) than the male patients with comparable ages (*p* = 0.629) and cirrhosis (*p* = 0.401) rates.

**Table 1:** Baseline characteristics of the patients with primary biliary cholangitis [mean+/−standard deviation/median (range)].

	All (n = 150)	Female (n = 128)	Male (n = 22)	*p* values
Age (yrs)	53.75 ± 11.66/53(25~80)	53.44 ± 10.88/53(25~79)	55.59 ± 15.61/52.50(25~80)	0.629
AMA (diluted titer)	564.1 ± 485.1/320(20~1280)	543.7 ± 480.3/320(20~1280)	665.8 ± 511.2/640(40~1280)	0.365
ANA (diluted titer)	723.4 ± 525.9/640(20~1280)	727.3 ± 528.4/640(20~1280)	690.0 ± 538.6/640(40~1280)	0.810
AST(U/L)	102.0 ± 87.45/85(14~823)	97.99 ± 64.17/86(14~488)	124.8 ± 167.9/74.5(25~823)	0.696
ALT(U/L)	105.1 ± 111.2/87(8~1177)	95.92 ± 66.61/85(8~435)	157.5 ± 237.7/98.5(35~1177)	0.249
AST/ALT	1.14 ± 0.64/0.93(0.29~4.45)	1.19 ± 0.67/1.00(0.29~4.45)	0.89 ± 0.40/0.76(0.42~1.75)	0.008*
Alk-P (U/L)	376.9 ± 241.7/323 (141~1345)	376.4 ± 234.1/325 (141~1341)	370.9 ± 288.2303 (141~1345)	0.795
r-GT (U/L)	309.2 ± 238.1/235(16~1365)	305.3 ± 244.5/229(16~1365)	330.5 ± 202.7/342(44~701)	0.390
Total bilirubin (mg/dL)	2.66 ± 4.89/1.2(0.3~46.4)	2.71 ± 5.26/1.1(0.3~46.4)	2.42 ± 1.71/1.8(0.5~6.5)	0.069
aFP (ng/mL)	3.88 ± 1.964/3(1~14)	3.84 ± 1.87/3(1~14)	4.13 ± 2.45/3(2~13)	0.798
Albumin (g/dL)	4.03 ± 0.60/4.1(1.9~5.3)	4.00 ± 0.59/4.1(1.9~5.2)	4.21 ± 0.61/3(2~13)	0.244
TC (mg/dL)	267.89 ± 94.16/248(89~610)	275.49 ± 96.55/258(89~610)	213.9 ± 51.1/208(144~343)	0.029*
Liver cirrhosis, n (%)	34 (22.7)	30 (23.4)	4 (18.2)	0.401
Hypertension, n (%)	10 (6.7)	7 (5.5)	3 (13.6)	0.194
Dyslipidemia, n (%)	7 (4.7)	6 (4.7)	1 (4.5)	0.738
Diabetes, n (%)	6 (4.0)	5 (3.9)	1 (4.5)	0.610
Hyperuricemia, n (%)	3 (2.0)	1 (0.8)	2 (9.1)	0.054
Autoimmune disease, n (%)	7 (4.8)	5 (4.0)	2 (9.5)	0.262

CI: confidence interval; AMA: antimitochondrial antibody; ANA: antinuclear antibody; AST: aspartate transaminase; ALT: alanine aminotransferase; Alk-p: alkaline phosphatase; rGT: gamma-glutamyltransferase; aFP: alpha fetoprotein; TC: total cholesterol; *p < 0.05.

### UDCA response

The UDCA responders accounted for 70.6% of all the enrolled PBC patients and for 28.6% and 87.8% in those with and without baseline cirrhosis, respectively. That is, those with baseline cirrhosis had a lower UDCA response rate (*p* = 0.003). However, higher UDCA response rates were noted in those with than those without baseline autoimmune diseases (85.7% vs. 40%, *p* = 0.023). The UDCA response rates were indifferent among those with and without other baseline extrahepatic events, including hypertension (*p* = 0.206), dyslipidemia (*p* = 0.363), diabetes (*p* = 0.609) and hyperuricemia (*p* = 0.674).

### Cumulative incidence of various events

**Figure 1:**
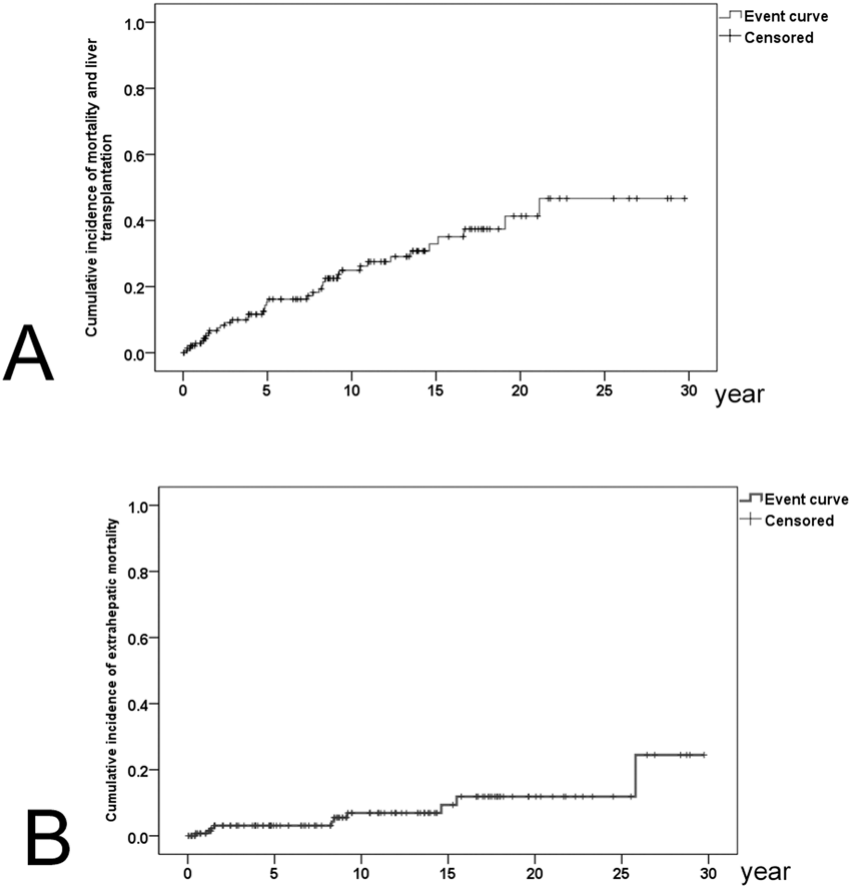
Cumulative incidences of all-cause mortality and liver transplantation (**A**) and extrahepatic (**B**) mortality in PBC patients.

**Figure 2:**
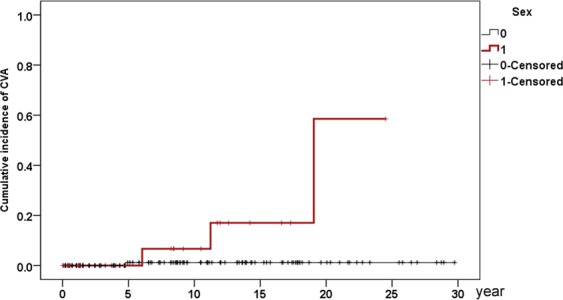
Cumulative incidence of incident CVA in PBC patients. Red line: male patients; black line: female patients.

With a follow-up period of up to 30 years, the cumulative incidences of various incident events were as follows (Table 2): all-cause mortality and liver transplantation (i.e., mortality for all causes and number of liver transplantations): 46.7% (16.2% for 5 years, 24.9% for 10 years, 32.9% for 15 years and 41.3% for 20 years) (Fig. 1A); extrahepatic mortality: 24.5% (3.1% for 5 years; 6.9% for 10 years; 9.4% for 15 years and 11.9% for 20 years) (Fig. 1B); EM: 8.1%; hypertension: 46.2%; dyslipidemia: 44.1%; diabetes: 30.6%; hyperuricemia: 11.2%; ACS: 3.1%; CVA: 8.9% (Fig. 2); autoimmune diseases: 16% (SS: 12.1%; SLE: 9.2%; RA:1.6%; autoimmune thyroid disease: 3.4%); and osteoporosis: 4.4% for 3 and 5 years and 20.9% for 30 years.

**Table 2:** Cumulative incidences of various incident extrahepatic events in patients with primary biliary cholangitis.

Events	5-year CI	10-year CI	15-year CI	20-year CI	Overall CI
All-cause mortality and liver transplantation	16.2%	24.9%	32.9%	41.3%	46.7%
Extrahepatic mortality	3.1%	6.9%	9.4%	11.9%	24.5%
Extrahepatic malignancies	1.1%	2.3%	8.1%	8.1%	8.1%
Hypertension	8.2%	12.9%	29.1%	39.5%	46.2%
Dyslipidemia	7.6%	19.8%	31.9%	38.5%	44.1%
Diabetes	8.8%	11.3%	18.4%	30.6%	30.6%
Hyperuricemia	1.1%	3.4%	7.4%	11.2%	11.2%
ACS	0%	1.1%	3.1%	3.1%	3.1%
CVA	1%	2.1%	3.9%	8.9%	8.9%
Autoimmune disease	7%	7%	10.7%	16%	16%
Osteoporosis	4.4%	8.6%	16%	20.9%	20.9%

CI: cumulative incidence; ACS: acute coronary syndrome; CVA: cerebrovascular accident.

Ten PBC patients died of extrahepatic causes, including 8 from sepsis subsequent to pneumonia (2 had baseline liver cirrhosis) or urinary tract infection, 1 from SLE and 1 from an uncertain cause. None of the 10 patients had HCC. Six PBC patients had incidents of EMs, including 3 colon cancers, 1 lung cancer, 1 cervical cancer and 1 brain cancer.

### Independent baseline factors of various incident extrahepatic events

**Figure 3:**
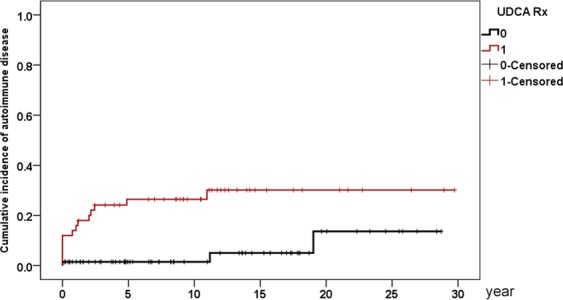
Cumulative incidence of autoimmune disease in PBC patients. Red line: UDCA responder; black line: UDCA suboptimal responder.

The independent factors for various incident extrahepatic events are as follows: (1) age (95% confidence interval (CI) of hazard ratio (HR):1.019–1.152; cut-off: 53 years, HR:6.499, 95% CI HR:1.323–31.935) and AFP (95% CI HR:1.002–1.058) with extrahepatic mortality (Supplementary Table 1); (2) age (95% CI HR: 1.022–1.225; cut-off: 57 years, HR: 5.948, 95% CI HR: 1.122–31.532) with incident EMs (Supplementary Table 2); (3) age (95% CI HR:1.012–1.083; cut-off: 53 years, HR: 3.124, 95% CI HR: 1.362–7.165) and hyperuricemia (95% HR: 1.701–37.06) with incident hypertension (Supplementary Table 3); (4) hypertension (95% CI HR: 1.417–24.19) with incident diabetes (Supplementary Table 4); (5) male gender (95% CI HR: 1.146–142.6) and hyperuricemia (95% CI HR: 1.14–355.4) with incident CVA (Supplementary Table 5 and Fig. 2); (6) UDCA response (95% CI HR: 1.049–24.33) with incident autoimmune disease (Supplementary Table 9 and Fig. 3); and (7) age (95% CI HR: 1.024–1.13; cut-off: 51 years; HR: 14.884, 95% CI HR:1.916–115.61) with incident osteoporosis (Supplementary Table 10). No independent factors could be identified for incident dyslipidemia, hyperuricemia and ACS (Supplementary Tables 6–8).

## Discussion

The key results of the current study are: (1) The female-to-male ratio was 5.82:1 for the 150 Taiwanese PBC patients. The females had higher AST/ALT ratios than the males. (2) The 30-year cumulative incidences of the various extrahepatic events ranged from 3.1% to 46.7%. Sepsis accounted for most of the extrahepatic mortality. (3) The baseline factors for various incident events are: age and AFP for extrahepatic mortality; age for EMs; age and hyperuricemia for hypertension; hypertension for diabetes; male gender and hyperuricemia for CVA; UDCA response for autoimmune disease; and age for osteoporosis.

The female-to-male ratio of 5.82:1 in the current study was less than 9–10:1, a ratio classically reported for PBC^5,38,39^. However, several population-based studies suggest an increasing male prevalence for PBC^8,40–44^, and the opposite trend was observed for the sample size and female-to-male ratio^44^. Thus, the percentage of males with PBC in most studies might be underestimated and highlights the importance of surveying the gender impact on the prognosis of PBC. The fact that the females had higher baseline AST/ALT ratios than the males suggests more advanced hepatic fibrosis^45^ despite comparable cirrhosis rate. While the higher cholesterol levels in the females might be subsequent to PBC-related liver disease severity or non-PBC-specific gender-dimorphic lipid metabolism^5^. The hypercholesteremia in female PBC patients did not alter their prognosis, as incident CVA was the only investigated extrahepatic event affected by gender, and male patients were the dominant victims.

Notably, although extrahepatic mortality (24.5%) accounts for more than 1/2 of all-cause mortality (46.7%) within a 30-year follow-up, it accounts for only 1/5 (3.1% vs. 16.2%), 1/4 (6.9% vs. 24.9%), 1/3 (9.4% vs. 32.9%), and less than 1/2 (11.9 vs. 41.3%) of the all-cause mortality within a 5-year, 10-year, 15-year and 20-year follow-up, respectively. That is, most 20-year mortalities were hepatic; however, the longer the follow-up, the higher the extrahepatic mortality-to-all-cause mortality ratio. In contrast to studies conducted in western countries^24,25^, the extrahepatic mortality (24.5%) of the PBC patients was not higher than that (25.71%^46^) of the general population in Taiwan. Both baseline age and AFP levels were associated with extrahepatic mortality, which was mainly caused by sepsis. Age-related remodeling of the immune system, termed immunosenescence, leads to infections that occur frequently in the elderly^47^. In addition to be a marker for HCC^48^, elevated AFP levels indicate hepatic regeneration subsequent to hepatic injury^49^ and induce immune escapes^50^. Both old age and high AFP levels thus might signify impaired immunity in the cases died of extrahepatic cause. Moreover, of the 8 patients who died of sepsis, 2 had baseline cirrhosis, an infection-susceptible disease^51^. Collectively, PBC is a potential immunocompromised disease that might threaten patients’ lives with the risk of sepsis.

The cumulative incidence (8.1%) of EM was within the reported range (4.5–15.7%)^10,11^. PBC increases the risk of bladder and breast cancers as found in a study conducted in Europe^52^. However, in the current study, colon cancer was the major EM, and none of the patients developed bladder or breast cancer. Whether any ethnic or environmental difference accounts for the discrepancy demands further investigation.

In a study conducted in the UK, the rates of ACS and CVA in PBC patients were comparable with controls^17^. However, in the current study, with the exception of dyslipidemia (7.6% for 5 years) and osteoporosis (4.4% for 3 years), the PBC-associated phenomena^2^, all cumulative incidences of metabolic diseases, including hypertension (8.2% for 5 years), diabetes (8.8% for 5 years), hyperuricemia (3.4% for 10 years), ACS (0% for 5 years), CVA (1% for 5 years), were lower than the general population. In Taiwanese adults, the 5-year cumulative incidence of hypertension, hyperglycemia, dyslipidemia^53^ and ACS^54^ was 24.2%, 20.04%, 7.3% and 1.5%, respectively; the 10-year cumulative incidences of hyperuricemia ranged from 11.5% to 18.1%^55^; the 3.3-year cumulative incidences of CVA ranged from 5.64% to 10.23%^56^; and the 3-year cumulative incidences of osteoporosis ranged from 2.1% to 3.3%^57^. In addition, although the incidence of autoimmune diseases in PBC patients was higher than in the general population of Taiwan (5-year cumulative incidence: 7% vs. 2.5–5%)^58^, it is much lower than in European PBC patients (30-year cumulative incidence: 16% vs. 61.2%)^59^, and different ethnicities might account for this difference.

Baseline age was also associated with incident EM, hypertension and osteoporosis. This finding was consistent with the notions that cancer and hypertension occur more frequently in the elderly due to immunosenescence^47^, aging leads to functional and mechanical changes in arteries^60^, and age is a major determinant of osteoporosis^61^. Based on the cut-off values of the baseline age for predicting osteoporosis (51 years), extrahepatic mortality (53 years), hypertension (53 years), and EM (57 years), PBC patients ≥51 years demand special attention for these complications. In addition, consistent with a previous study^62^, baseline hypertension was associated with incident diabetes. Although more male than female Chinese PBC patients had cardiac involvement^63^, we did not find any gender-dimorphism in the ACS risk. Instead, male gender and baseline hyperuricemia were positive factors for incident CVA. Consistently, previous studies showed that stroke incidence was higher in men than in women^64^, and gout is associated with stroke risk^65^.

In contrast to a previous report^7^, we did not find any connection between extrahepatic autoimmune diseases and EM. Paradoxically, a favorable UDCA response was associated with incident autoimmune diseases. Some UDCA inadequate responders died too early to acquire autoimmune diseases, which might account for this finding. However, a higher UDCA response rate was also noted in those with than those without baseline autoimmune diseases. Furthermore, most UDCA responders acquired autoimmune diseases within the first 5 years of follow-up, while the UDCA inadequate responder acquired autoimmune diseases with a time lag of 15 years approximately (Fig. 3). If patients prone to developing autoimmune diseases are primed to be better responders to UDCA remains elusive, further investigation is required.

The strength of the study is the long follow-up with a comprehensive survey of extrahepatic events, while the retrospective nature is the main limitation to elucidate the associated biases including selection bias.

Taken together, in this Taiwanese PBC cohort, a lower female-to-male ratio than expected was noted, and most extrahepatic complications were less frequent than the general population. Gender dimorphism was only evident in incident CVA. The longer the follow-up, the higher the extrahepatic-to-all-cause mortality ratio. Most extrahepatic mortality resulted from sepsis. PBC patients ≥51 years warrant special attention for osteoporosis, extrahepatic mortality, EM and hypertension. The positive link between favorable UDCA response and incident autoimmune diseases warrants further investigation.

## Supplementary information

Supplementary Tables 1–10

## Data Availability

The datasets generated during and/or analyzed during the current study are available from the corresponding author on reasonable request.
