# Supplementary material for: The extrahepatic events of Asian patients with primary biliary cholangitis: A 30-year cohort study
Source: Sci Rep. 2019 May 20;9:7577. doi: 10.1038/s41598-019-44081-x (PMC6527707; doi:10.1038/s41598-019-44081-x)
Supplement: Supplementary file 1 — Supplementary Tables 1–10 [file 41598_2019_44081_MOESM1_ESM.doc]

**The extrahepatic events of Asian patients with primary biliary cholangitis: A 30-year cohort study**

**Cheng-Yu Lin, Ya-Ting Cheng, Ming-Ling Chang, Rong-Nan Chien**

**Supplementary Table 1. The independent baseline factors for incident extrahepatic mortality of the patients with primary biliary cholangitis.**

|  | Univariate |  | Multivariate |  |
| --- | --- | --- | --- | --- |
|  | 95% CI of HR (HR) | *p* values | 95% CI of HR (HR) | *p* values |
| Sex (M) | 0.082~5.593 (0.644) | 0.644 |  |  |
| Age (yrs) | 1.025~1.053 (1.087) | 0.006 | 1.019~1.152 (1.083 ) | 0.011 |
| AMA (titer) | 0.999~1.002 (1.000) | 0.779 |  |  |
| ANA (titer) | 0.999~1.002 (1.000) | 0.629 |  |  |
| AST(U/L) | 0.99~1.007 (0.999) | 0.739 |  |  |
| ALT(U/L) | 0.982~1.005 (0.994) | 0.28 |  |  |
| Alk-P (U/L) | 0.994~1.001 (0.998) | 0.201 |  |  |
| r-GT (U/L) | 0.989~1.001 (0.995) | 0.11 |  |  |
| Bili (t) (mg/dL) | 0.851~1.398 (1.091) | 0.491 |  |  |
| aFP (ng/mL) | 1.003~1.056 (1.029) | 0.031 | 1.002~1.058 (1.03 ) | 0.036 |
| Albumin (g/dL) | 0.194~0.967(0.433) | 0.041 | 0.226~1.173 (0.515) | 0.114 |
| TC (mg/dL) | 0.988~1.005 (0.997) | 0.426 |  |  |
| LC (yes) | 0.069~4.349 (0.549) | 0.57 |  |  |
| H/T (yes) | 0.17~10.879 (1.358) | 0.773 |  |  |
| DL (yes) | 0~1689.0 (0.046) | 0.637 |  |  |
| DM (yes) | 0~1.37 (0.047) | 0.804 |  |  |
| HU (yes) | 0~4.5 (0.048) | 0.796 |  |  |
| AI (yes) | 0~2352 (0.048) | 0.737 |  |  |
| UDCA Rx (yes) | 0.472~8.402 (1.991) | 0.391 |  |  |

CI: confidence interval; HR: hazard ratio; AMA: antimitochondrial antibody; ANA: antinuclear antibody; AST: aspartate transaminase; ALT: alanine aminotransferase; Alk-p: alkaline phosphatase ; rGT: gamma-glutamyltransferase ; Bili(t): total bilirubin; aFP: alpha fetoprotein;TC: total cholesterol; LC: liver cirrhosis; H/T: hypertension; DL: dyslipidemia; DM: diabetes; HU: hyperuricemia; AI: autoimmune disease; UDCA Rx: ursodeoxycholic acid response

**Supplementary Table 2. The independent baseline factors for incident extrahepatic malignancies of the patients with primary biliary cholangitis.**

|  | Univariate |  | Multivariate |  |
| --- | --- | --- | --- | --- |
|  | 95% CI of HR (HR) | *p* values | 95% CI of HR (HR) | *p* values |
| Sex (M) | 0.155~12.446(1.388) | 0.770 |  |  |
| Age (yrs) | 1.021~1.184(1.094) | 0.024 | 1.022~1.225 (1.119) | 0.015 |
| AMA (titer) | 1.000~1.004(1.002) | 0.058 |  |  |
| ANA (titer) | 0.999~1.002(1.001) | 0.525 |  |  |
| AST(U/L) | 1.001~1.008(1.004) | 0.019 | 0.982~1.022(1.002) | 0.873 |
| ALT(U/L) | 1.001~1.005(1.003) | 0.013 | 0.989~1.016(1.003) | 0.702 |
| Alk-P (U/L) | 0.993~1.003(0.998) | 0.381 |  |  |
| r-GT (U/L) | 0.981~1.002(0.991) | 0.102 |  |  |
| Bili (t) (mg/dL) | 0.762~1.896(1.202) | 0.429 |  |  |
| aFP (ng/mL) | 0.992~1.726(1.309) | 0.057 |  |  |
| Albumin (g/dL) | 0.107~2.731(0.541) | 0.457 |  |  |
| TC (mg/dL) | 0.953~1.007(0.980) | 0.147 |  |  |
| LC (yes) | 0.148~11.916(1.330) | 0.799 |  |  |
| H/T (yes) | 0~105741.365(0.044) | 0.677 |  |  |
| DL (yes) | 0~1.594 (0.049) | 0.944 |  |  |
| DM (yes) | 0~2722395.12(0.046) | 0.736 |  |  |
| HU (yes) | 0~1.594 (0.049) | 0.944 |  |  |
| AI (yes) | 0~7703 (0.045) | 0.716 |  |  |
| UDCA Rx (yes) | 0.53~42.85 (4.765) | 0.164 |  |  |

CI: confidence interval; HR: hazard ratio; AMA: antimitochondrial antibody; ANA: antinuclear antibody; AST: aspartate transaminase; ALT: alanine aminotransferase; Alk-p: alkaline phosphatase ; rGT: gamma-glutamyltransferase ; Bili(t): total bilirubin; aFP: alpha fetoprotein;TC: total cholesterol; LC: liver cirrhosis; H/T: hypertension; DL: dyslipidemia; DM: diabetes; HU: hyperuricemia; AI: autoimmune disease; UDCA Rx: ursodeoxycholic acid response

**Supplementary Table 3. The independent baseline factors for incident hypertension of the patients with primary biliary cholangitis.**

|  | Univariate |  | Multivariate |  |
| --- | --- | --- | --- | --- |
|  | 95% CI of HR (HR) | *p* values | 95% CI of HR (HR) | *p* values |
| Sex (M) | 0.666~4.802(1.788) | 0.249 |  |  |
| Age (yrs) | 1.012~1.083(1.047) | 0.007 | 1.012~1.083(1.047) | 0.007 |
| AMA (titer) | 1.000~1.002(1.001) | 0.155 |  |  |
| ANA (titer) | 0.999~1.000(1.000) | 0.305 |  |  |
| AST(U/L) | 0.999~1.004(1.002) | 0.292 |  |  |
| ALT(U/L) | 0.999~1.003(1.001)) | 0.442 |  |  |
| Alk-P (U/L) | 0.997~1.001(0.999) | 0.176 |  |  |
| r-GT (U/L) | 0.997~1.002(1.000) | 0.914 |  |  |
| Bili (t) (mg/dL) | 0.720~1.310(0.971) | 0.848 |  |  |
| aFP (ng/mL) | 0.939~1.406(1.149) | 0.178 |  |  |
| Albumin (g/dL) | 0.302~2.062(0.790) | 0.630 |  |  |
| TC (mg/dL) | 0.993~1.005(0.999) | 0.678 |  |  |
| LC (yes) | 0.392~3.340(1.144) | 0.806 |  |  |
| H/T (yes) | NA |  |  |  |
| DL (yes) | 0~4615 (0.048) | 0.666 |  |  |
| DM (yes) | 0~3389 (0.048) | 0.770 |  |  |
| HU (yes) | 2.305~49.37 (10.67) | 0.002 | 1.701~37.06(7.94) | 0.008 |
| AI (yes) | 0~202.2(0.046) | 0.472 |  |  |
| UDCA Rx (yes) | 0.45~2.421 (1.044) | 0.921 |  |  |

CI: confidence interval; HR: hazard ratio; AMA: antimitochondrial antibody; ANA: antinuclear antibody; AST: aspartate transaminase; ALT: alanine aminotransferase; Alk-p: alkaline phosphatase ; rGT: gamma-glutamyltransferase ; Bili(t): total bilirubin; aFP: alpha fetoprotein;TC: total cholesterol; LC: liver cirrhosis; NA: not assessible; H/T: hypertension; DL: dyslipidemia; DM: diabetes; HU: hyperuricemia; AI: autoimmune disease; UDCA Rx: ursodeoxycholic acid response

**Supplementary Table 4**. The independent baseline factors for incident diabetes of the patients with primary biliary cholangitis.

|  | Univariate |  | Multivariate |  |
| --- | --- | --- | --- | --- |
|  | 95% CI of HR (HR) | *p* values | 95% CI of HR (HR) | *p* values |
| Sex (M) | 0.037~2.074(0.278) | 0.212 |  |  |
| Age (yrs) | 1.006~1.083(1.044) | 0.023 | 0.9821.074(1.027) | 0.238 |
| AMA (titer) | 0.999~1.001(1.000) | 0.910 |  |  |
| ANA (titer) | 0.998~1.000(0.999) | 0.178 |  |  |
| AST(U/L) | 0.971~0.995(0.983) | 0.008 | 0.984~1.008(0.996) | 0.541 |
| ALT(U/L) | 0.982~1.000(0.991) | 0.042 |  |  |
| Alk-P (U/L) | 0.992~0.999*0.996) | 0.010 | 0.992~1.000(0.996) | 0.059 |
| r-GT (U/L) | 0.999~1.003(1.001) | 0.542 |  |  |
| Bili (t) (mg/dL) | 0.669~1.263(0.919) | 0.603 |  |  |
| aFP (ng/mL) | 0.969~1.387(1.159) | 0.106 |  |  |
| Albumin (g/dL) | 0.240~1.861(0.669) | 0.441 |  |  |
| TC (mg/dL) | 0.990~1.004(0.997) | 0.375 |  |  |
| LC (yes) | 0.447~3.983(1.334) | 0.606 |  |  |
| H/T (yes) | 1.189~14.981(4.221) | 0.026 | 1.417~24.19 (5.856) | 0.015 |
| DL (yes) | 0.186~10.583(1.402) | 0.743 |  |  |
| DM (yes) | NA |  |  |  |
| HU (yes) | 0~21582857.5(0.0.48) | 0.766 |  |  |
| AI (yes) | 0.777~15.003(3.415) | 0.104 |  |  |
| UDCA Rx (yes) | 1.25~8.49(3.255) | 0.016 | 0.888~8.144 (2.689) | 0.08 |

CI: confidence interval; HR: hazard ratio; AMA: antimitochondrial antibody; ANA: antinuclear antibody; AST: aspartate transaminase; ALT: alanine aminotransferase; Alk-p: alkaline phosphatase ; rGT: gamma-glutamyltransferase ; Bili(t): total bilirubin; aFP: alpha fetoprotein;TC: total cholesterol; LC: liver cirrhosis; H/T: hypertension; DL: dyslipidemia; DM: diabetes; NA: not assessible; HU: hyperuricemia; AI: autoimmune disease; UDCA Rx: ursodeoxycholic acid response

**Supplementary Table 5**. The independent baseline factors for incident CVA of the patients with primary biliary cholangitis.

|  | Univariate |  | Multivariate |  |
| --- | --- | --- | --- | --- |
|  | 95% CI of HR (HR) | *p* values | 95% CI of HR (HR) | *p* values |
| Sex (M) | 1.858~175.258(18.047) | 0.013 | 1.145~142.6(12.782) | 0.038 |
| Age (yrs) | 0.952~1.120(1.032) | 0.441 |  |  |
| AMA (titer) | 0.999~1.004(1.001) | 0.251 |  |  |
| ANA (titer) | 0.996~1.002(0.999) | 0.439 |  |  |
| AST(U/L) | 0.980~1.013(0.996) | 0.667 |  |  |
| ALT(U/L) | 0.976~1.011(0.993) | 0.453 |  |  |
| Alk-P (U/L) | 0.995~1.004(0.999) | 0.701 |  |  |
| r-GT (U/L) | 0.995~1.005(1.000) | 0.947 |  |  |
| Bili (t) (mg/dL) | 0.384~2.152(0.909) | 0.828 |  |  |
| aFP (ng/mL) | 0.202~1.846(0.611) | 0.383 |  |  |
| Albumin (g/dL) | 0.103~5.475(0.750) | 0.777 |  |  |
| TC (mg/dL) | 0.970~1.011(0.990) | 0.347 |  |  |
| LC (yes) | 0.212~19.857(2.052) | 0.535 |  |  |
| H/T (yes) | 0.547~66.720(6.042) | 0.142 |  |  |
| DL (yes) | 0~1.233(0.046) | 0.802 |  |  |
| DM (yes) | 0~8.215 (0.049) | 0.922 |  |  |
| HU (yes) | 5.812~1486.60(92.95) | 0.001 | 1.146~355.3 (20.179) | 0.040 |
| AI (yes) | 0~8035 (0.045) | 0.776 |  |  |
| UDCA Rx (yes) | 0.039~3.65 (0.377) | 0.399 |  |  |

CI: confidence interval; HR: hazard ratio; AMA: antimitochondrial antibody; ANA: antinuclear antibody; AST: aspartate transaminase; ALT: alanine aminotransferase; Alk-p: alkaline phosphatase ; rGT: gamma-glutamyltransferase ; Bili(t): total bilirubin; aFP: alpha fetoprotein;TC: total cholesterol; LC: liver cirrhosis; H/T: hypertension; DL: dyslipidemia; DM: diabetes; HU: hyperuricemia; AI: autoimmune disease; UDCA Rx: ursodeoxycholic acid response

**Supplementary Table 6. The independent baseline factors for incident dyslipidemia of the patients with primary biliary cholangitis.**

|  | Univariate |  | Multivariate |  |
| --- | --- | --- | --- | --- |
|  | 95% CI of HR (HR) | *p* values | 95% CI of HR (HR) | *p* values |
| Sex (M) | 0.089~1.583(0.375) | 0.182 |  |  |
| Age (yrs) | 0.987~1.048(1.017) | 0.271 |  |  |
| AMA (titer) | 0.999~1.001(1.000) | 0.955 |  |  |
| ANA (titer) | 0.998~1.000(0.999) | 0.124 |  |  |
| AST(U/L) | 0.996~1.004(1.000) | 0.954 |  |  |
| ALT(U/L) | 0.997~1.003(1.000) | 0.918 |  |  |
| Alk-P (U/L) | 0.998~1.001(0.999) | 0.398 |  |  |
| r-GT (U/L) | 0.998~1.002(1.000) | 0.875 |  |  |
| Bili (t) (mg/dL) | 0.531~1.122(0.772) | 0.175 |  |  |
| aFP (ng/mL) | 0.829~1.285(1.032) | 0.776 |  |  |
| Albumin (g/dL) | 0.329~1.856(0.760) | 0.520 |  |  |
| TC (mg/dL) | 0.998~1.007(1.003) | 0.219 |  |  |
| LC (yes) | 0.241~2.667(0.801) | 0.718 |  |  |
| H/T (yes) | 0.916~10.625(3.119) | 0.069 |  |  |
| DL (yes) | NA |  |  |  |
| DM (yes) | 0.285~16.403(2.161) | 0.456 |  |  |
| HU (yes) | 0.979~63.639(7.982) | 0.052 |  |  |
| AI (yes) | 0.339~6.075(1.435) | 0.624 |  |  |
| UDCA Rx (yes) | 0.679~3.163(1.466) | 0.33 |  |  |

CI: confidence interval; HR: hazard ratio; AMA: antimitochondrial antibody; ANA: antinuclear antibody; AST: aspartate transaminase; ALT: alanine aminotransferase; Alk-p: alkaline phosphatase ; rGT: gamma-glutamyltransferase ; Bili(t): total bilirubin; aFP: alpha fetoprotein;TC: total cholesterol; LC: liver cirrhosis; H/T: hypertension; DL: dyslipidemia; NA: not assessible; DM: diabetes; HU: hyperuricemia; AI: autoimmune disease; UDCA Rx: ursodeoxycholic acid response

**Supplementary Table 7**. The independent baseline factors for incident hyperuricemia of the patients with primary biliary cholangitis.

|  | Univariate |  | Multivariate |  |
| --- | --- | --- | --- | --- |
|  | 95% CI of HR (HR) | *p* values | 95% CI of HR (HR) | *p* values |
| Sex (M) | 0.546~16.693(3.020) | 0.205 |  |  |
| Age (yrs) | 0.912~1.055(0.981) | 0.602 |  |  |
| AMA (titer) | 0.998~1.002(1.000) | 0.869 |  |  |
| ANA (titer) | 1.000~1.003(1.001) | 0.151 |  |  |
| AST(U/L) | 1.001~1.007(1.004) | 0.014 | 0.972~1.024(0.998) | 0.859 |
| ALT(U/L) | 1.001~1.005(1.003) | 0.007 | 0.983~1.027(1.005) | 0.674 |
| Alk-P (U/L) | 0.993~1.002(0.997) | 0.304 |  |  |
| r-GT (U/L) | 0.992~1.004(0.998) | 0.538 |  |  |
| Bili (t) (mg/dL) | 0.653~1.854(1.100) | 0.720 |  |  |
| aFP (ng/mL) | 1.027~1.769(1.348) | 0.031 | 0.459~2.101(1.020) | 0.958 |
| Albumin (g/dL) | 0.207~10.205(1.452) | 0.707 |  |  |
| TC (mg/dL) | 0.992~1.018(1.005) | 0.473 |  |  |
| LC (yes) | 0.124~9.188(1.069) | 0.952 |  |  |
| H/T (yes) | 0~1223 (0.044) | 0.680 |  |  |
| DL (yes) | 0~1927 (0.046) | 0.731 |  |  |
| DM (yes) | 0~3.036 (0.049) | 0.902 |  |  |
| HU (yes) | NA |  |  |  |
| AI (yes) | 0~256266.746(0.046) | 0.697 |  |  |
| UDCA Rx (yes) | 0.234~5.83 (1.168) | 0.85 |  |  |

CI: confidence interval; HR: hazard ratio; AMA: antimitochondrial antibody; ANA: antinuclear antibody; AST: aspartate transaminase; ALT: alanine aminotransferase; Alk-p: alkaline phosphatase ; rGT: gamma-glutamyltransferase ; Bili(t): total bilirubin; aFP: alpha fetoprotein;TC: total cholesterol; LC: liver cirrhosis; H/T: hypertension; DL: dyslipidemia; DM: diabetes; HU: hyperuricemia; NA: not assessible; AI: autoimmune disease; UDCA Rx: ursodeoxycholic acid response

**Supplementary Table 8. The independent baseline factors for incident ACS of the patients with primary biliary cholangitis.**

|  | Univariate |  | Multivariate |  |
| --- | --- | --- | --- | --- |
|  | 95% CI of HR (HR) | *p* values | 95% CI of HR (HR) | *p* values |
| Sex (M) | 0.377~98.614(6.096) | 0.203 |  |  |
| Age (yrs) | 0.985~1.427(1.185) | 0.072 |  |  |
| AMA (titer) | 0.998~1.004(1.001) | 0.573 |  |  |
| ANA (titer) | 0.997~1.004(1.001) | 0.746 |  |  |
| AST(U/L) | 0.936~1.026(0.980) | 0.387 |  |  |
| ALT(U/L) | 0.954~1.020(0.987) | 0.431 |  |  |
| Alk-P (U/L) | 0.989~1.006(0.997) | 0.534 |  |  |
| r-GT (U/L) | 1.001~1.007(1.004) | 0.006 | 0.997~1.011(1.004) | 0.276 |
| Bili (t) (mg/dL) | 0.748~2.544(1.380) | 0.302 |  |  |
| aFP (ng/mL) | 0.551~2.224(1.106) | 0.776 |  |  |
| Albumin (g/dL) | 0.005~3.576(0.136) | 0.232 |  |  |
| TC (mg/dL) | 0.978~1.014(0.996) | 0.643 |  |  |
| LC (yes) | 0.325~84.136(5.228) | 0.243 |  |  |
| H/T (yes) | 0~3.3515(932430.154) | 0.945 |  |  |
| DL (yes) | 1.144~297.886(18.549) | 0.040 | 0.001~1130.092(1.157) | 0.967 |
| DM (yes) | 0~1.5945(0.049) | 0.994 |  |  |
| HU (yes) | 0~14611(1.000) | 1.000 |  |  |
| AI (yes) | 0~1.303 (0.046) | 0.818 |  |  |
| UDCA Rx (yes) | 0.001~7135(76.8) | 0.457 |  |  |

CI: confidence interval; HR: hazard ratio; AMA: antimitochondrial antibody; ANA: antinuclear antibody; AST: aspartate transaminase; ALT: alanine aminotransferase; Alk-p: alkaline phosphatase ; rGT: gamma-glutamyltransferase ; Bili(t): total bilirubin; aFP: alpha fetoprotein;TC: total cholesterol; LC: liver cirrhosis; H/T: hypertension; DL: dyslipidemia; DM: diabetes; HU: hyperuricemia; AI: autoimmune disease; UDCA Rx: ursodeoxycholic acid response

**Supplementary Table 9. The independent baseline factors for incident autoimmune diseases of the patients with primary biliary cholangitis.**

|  | Univariate |  | Multivariate |  |
| --- | --- | --- | --- | --- |
|  | 95% CI of HR (HR) | *p* values | 95% CI of HR (HR) | *p* values |
| Sex (M) | 0.080~4.915(0.828) | 0.658 |  |  |
| Age (yrs) | 0.947~1.044(0.994) | 0.814 |  |  |
| AMA (titer) | 0.999~1.002(1.000) | 0.626 |  |  |
| ANA (titer) | 0.998~1.001(0.999) | 0.4380 |  |  |
| AST(U/L) | 0.998~1.006(1.002) | 0.411 |  |  |
| ALT(U/L) | 0.995~1.005(1.000) | 0.992 |  |  |
| Alk-P (U/L) | 0.994~1.001(0.997) | 0.156 |  |  |
| r-GT (U/L) | 0.992~1.002(0.997) | 0.207 |  |  |
| Bili (t) (mg/dL) | 0.236~1.485(0.592) | 0.264 |  |  |
| aFP (ng/mL) | 0.141~1.074(0.389) | 0.068 |  |  |
| Albumin (g/dL) | 0.174~2.474(0.656) | 0.534 |  |  |
| TC (mg/dL) | 0.990~1.008(0.999) | 0.809 |  |  |
| LC (yes) | 0~26.035(0.036) | 0.322 |  |  |
| H/T (yes) | 0~1447.277(0.044) | 0.556 |  |  |
| DL (yes) | 0~18298.311(0.046) | 0.640 |  |  |
| DM (yes) | 0.691~47.108(5.707) | 0.106 |  |  |
| HU (yes) | 0~1548 (0.049) | 0.806 |  |  |
| AI (yes) | NA |  |  |  |
| UDCA Rx (yes) | 1.049~24.33(5.047) | 0.044 |  |  |

CI: confidence interval; HR: hazard ratio; AMA: antimitochondrial antibody; ANA: antinuclear antibody; AST: aspartate transaminase; ALT: alanine aminotransferase; Alk-p: alkaline phosphatase ; rGT: gamma-glutamyltransferase ; Bili(t): total bilirubin; aFP: alpha fetoprotein;TC: total cholesterol; LC: liver cirrhosis; H/T: hypertension; DL: dyslipidemia; DM: diabetes; HU: hyperuricemia; AI: autoimmune disease; NA: not assessible; UDCA Rx: ursodeoxycholic acid response

**Supplementary Table 10. The independent baseline factors for incident osteoporosis of the patients with primary biliary cholangitis.**

|  | Univariate |  | Multivariate |  |
| --- | --- | --- | --- | --- |
|  | 95% CI of HR (HR) | *p* values | 95% CI of HR (HR) | *p* values |
| Sex (M) | 0.054~3.113 (0.409) | 0.388 |  |  |
| Age (yrs) | 1.026~1.119 (1.072) | 0.002 | 1.024~1.13 (1.076) | 0.004 |
| AMA (titer) | 0.999~1.001 (1.000) | 0.863 |  |  |
| ANA (titer) | 0.997~1.000 (0.998) | 0.057 |  |  |
| AST(U/L) | 0.979~1.102 (0.995) | 0.575 |  |  |
| ALT(U/L) | 0.999~1.004 (1.002) | 0.254 |  |  |
| Alk-P (U/L) | 0.993~1.000 (0.996) | 0.029 | 0.993~1.00 (0.996) | 0.061 |
| r-GT (U/L) | 0.999~1.003 (1.001) | 0.91 |  |  |
| Bili (t) (mg/dL) | 0.612~1.365 (0.914) | 0.662 |  |  |
| aFP (ng/mL) | 0.847~1.431 (1.101) | 0.473 |  |  |
| Albumin (g/dL) | 0.288~3.386 (0.987) | 0.983 |  |  |
| TC (mg/dL) | 0.985~1.004 (0.995) | 0.288 |  |  |
| LC (yes) | 0.308~3.892 (1.094) | 0.89 |  |  |
| H/T (yes) | 0.498~9.491 (2.23) | 0.294 |  |  |
| DL (yes) | 0.19~11.15 (1.457) | 0.717 |  |  |
| DM (yes) | 0.551~38.84 (4.445) | 0.161 |  |  |
| HU (yes) | 0~1.437 (0.049) | 0.786 |  |  |
| AI (yes) | 0.193~11.29 (1.475) | 0.708 |  |  |
| UDCA Rx (yes) | 0.889~9.067 (2.838) | 0.078 |  |  |

CI: confidence interval; HR: hazard ratio; AMA: antimitochondrial antibody; ANA: antinuclear antibody; AST: aspartate transaminase; ALT: alanine aminotransferase; Alk-p: alkaline phosphatase ; rGT: gamma-glutamyltransferase ; Bili(t): total bilirubin; aFP: alpha fetoprotein;TC: total cholesterol; LC: liver cirrhosis; H/T: hypertension; DL: dyslipidemia; DM: diabetes; HU: hyperuricemia; AI: autoimmune disease; UDCA Rx: ursodeoxycholic acid response
